# 824. In Vivo Models of Frostbite: A Systematic Review of Interventions and Outcome Metrics

**DOI:** 10.1093/jbcr/irag033.263

**Published:** 2026-04-06

**Authors:** Christopher J Fedor, Alexa Rivera del Rio Hernandez, Kelson Huynh, Hunter Waltermire, Francesco M Egro

**Affiliations:** University of Pittsburgh Medical Center, Department of Plastic Surgery, Pittsburgh, PA; University of Pittsburgh Medical Center, Department of Plastic Surgery, Pittsburgh, PA; University of Pittsburgh Medical Center, Department of Plastic Surgery, Pittsburgh, PA; University of Pittsburgh, School of Medicine, Pittsburgh, PA; University of Pittsburgh Medical Center, Pittsburgh, PA

## Abstract

**Introduction:**

Frostbite is a severe cold-induced injury characterized by ischemia, necrosis, and long-term morbidity. Standardizing experimental models is difficult because frostbite arises from overlapping pathophysiologic phases and is influenced by freezing rate, tissue type, thaw-refreeze cycles, and anatomical variability. Models often yield inconsistent depth and extent of injury, while clinical frostbite is inherently heterogeneous and long-term sequelae such as neurovascular damage and pain are challenging to reproduce. These factors complicate comparison across studies and highlight the need to evaluate models of frostbite wound healing and regeneration.

**Methods:**

A systematic literature search was performed to identify in vivo frostbite studies reporting wound healing or regeneration endpoints. Extracted variables included animal species, number and weight of animals, method of frostbite induction, anatomical site of injury, therapeutic interventions, and outcome measures.

**Results:**

Twenty-five studies were identified. Rats (13) and mice (10) were most frequent, with fewer rabbit models (3), which have not been employed in recent literature. On average, 41 animals (range: 16–96) were used per study. Reported weights ranged from 18–25 g in mice, 174–300 g in rats, and 3–3.5 kg in rabbits. Frostbite was most often induced by contact freezing, with nine studies each using plates at −80°C (dry ice) or − 196°C (liquid nitrogen). Less common methods included ethanol–water immersion (n = 3), liquid nitrogen immersion (n = 1), nitrogen gas jets (n = 1), and one low-temperature exposure model. Therapeutics grouped into natural/herbal extracts (n = 5), biological/cell-based (n = 4), synthetic/nanomaterials (n = 6), conventional pharmacologics (n = 5), and physical/energy-based modalities (n = 2); one study used autologous tissue transfer (split thickness skin graft and flap). Outcomes centered on macroscopic healing (wound area, contraction, gross scores) and histopathology. H&E and Masson’s trichrome were most common, often with semi-quantitative scoring of necrosis, inflammation, granulation, fibroblast maturation, and re-epithelialization. Few studies added immunohistochemistry or molecular assays for angiogenesis, apoptosis, or inflammatory markers.

**Conclusions:**

In vivo frostbite models are heterogeneous but consistently emphasize wound closure and histopathological recovery as core outcomes. Rodents dominate due to availability and cost, while rabbits allow multiple injuries per animal and larger species may better replicate human responses. No single model can yet be recommended; selection should align with study aims, feasibility, transferability, and ethics.

**Applicability of Research to Practice:**

There is great need for cold injury research. Because few studies discuss refined in vivo models, we aimed to outline what has been achieved in animal models, describe current approaches, and summarize their strengths and weaknesses.

**Funding for the study:**

N/A.

